# First Evidence for Wollemi Pine-type Pollen (*Dilwynites*: Araucariaceae) in South America

**DOI:** 10.1371/journal.pone.0069281

**Published:** 2013-07-19

**Authors:** Mike Macphail, Raymond J. Carpenter, Ari Iglesias, Peter Wilf

**Affiliations:** 1 Department of Archaeology and Natural History, College of Asia and the Pacific, Australian National University, Canberra, Australian Capital Territory, Australia; 2 School of Earth and Environmental Sciences, University of Adelaide, South Australia, Australia; 3 División Palaeobotánica, Facultad de Ciencias Naturales y Museo, Universidad Nacional de La Plata, Paseo del Bosque s/n, La Plata, Argentina; 4 Consejo Nacional de Investigaciones Científicas y Técnicas, Argentina; 5 Department of Geosciences, Pennsylvania State University, University Park, Pennsylvania, United States of America; The Pennsylvania State University, United States of America

## Abstract

We report the first fossil pollen from South America of the lineage that includes the recently discovered, extremely rare Australian Wollemi Pine, *Wollemia nobilis* (Araucariaceae). The grains are from the late Paleocene to early middle Eocene Ligorio Márquez Formation of Santa Cruz, Patagonia, Argentina, and are assigned to *Dilwynites*, the fossil pollen type that closely resembles the pollen of modern *Wollemia* and some species of its Australasian sister genus, *Agathis*. *Dilwynites* was formerly known only from Australia, New Zealand, and East Antarctica. The Patagonian *Dilwynites* occurs with several taxa of Podocarpaceae and a diverse range of cryptogams and angiosperms, but not *Nothofagus*. The fossils greatly extend the known geographic range of *Dilwynites* and provide important new evidence for the Antarctic region as an early Paleogene portal for biotic interchange between Australasia and South America.

## Introduction

The Southern Hemisphere monkey-puzzle tree family, Araucariaceae, was long believed to comprise two living genera: *Araucaria* Juss., with about 19 species endemic to the southwest Pacific and South America, and *Agathis* Salisb., with about 20 species distributed from Sumatra to New Zealand but absent in South America. Remarkably, a third araucarian genus was discovered in 1994 in New South Wales, Australia, whose sole species is *Wollemia nobilis* W.G. Jones, K.D. Hill & J.M. Allen, common name Wollemi Pine [1].

With fewer than 40 adult specimens known to survive in the wild, *W*. *nobilis* is one of the world’s rarest trees. Adding to the spectacular nature of the discovery was the location of the stands, in a remote gorge within 150 km of Sydney, Australia’s largest city; the large stature of the trees (up to 40 m tall); and the apparent similarity of the foliage to that of the Jurassic species “*Agathis*” *jurassica* M.E. White [2] and the Cretaceous to early Cenozoic genus *Araucarioides* [3]–[6]. So far, none of the similar macrofossils has been convincingly demonstrated to belong to *Wollemia* [6], [7], and indeed “*A*.” *jurassica* differs from foliage of *Wollemia* in details of venation, leaf arrangement and leaf shape [8]. However, the presumed close relationship quickly led to *W*. *nobilis* being given the status of a “living fossil from the age of dinosaurs” in the popular press (e.g. [9]).

In contrast, once pollen was made available, it was quickly recognized that the *Wollemia* clade had a well-established fossil history provided by the morphogenus *Dilwynites* W.K. Harris [10], comprising *D. granulatus* W.K. Harris and *D. tuberculatus* W.K. Harris [6], [11], [12]. For example, in southern Australia, *D*. *granulatus*, the morphospecies that most closely resembles modern *Wollemia* pollen, can be traced back as far as the Turonian Age (89.8 to 93.9 Ma) of the Late Cretaceous [11]–[13]. So far, *Dilwynites* has been identified in Paleogene to Neogene deposits of western, central, and northern Australia (references in [14]), in Cretaceous to Neogene deposits of New Zealand [15], and in late Eocene deposits of East Antarctica [16]. However, apart from a possible record from the Paleogene of Seymour Island [17], no *Dilwynites* pollen has previously been recognized from West Gondwana.

Since 2000, the first author has also recognized that at least one species of *Agathis* produces pollen that is morphologically consistent with *Dilwynites*, and thus the nearest extant relatives of the plants that produced *Dilwynites* pollen are best regarded as both *Wollemia* and *Agathis* (e.g., [14]). This observation is consistent with the results of many recent molecular studies indicating that *Wollemia* and *Agathis* are sister taxa [18]–[23], along with several characteristics of the seed cones of the two genera that may be synapomorphic, especially the condition of the seeds being winged and nearly free from the fused bract and scale [24]. By contrast, in *Araucaria*, the seeds are embedded in the bract/scale complex.

We here present microfossil evidence that araucarians producing *Dilwynites* pollen were growing in southern Patagonia during the late Paleocene to early middle Eocene. This discovery greatly augments evidence for the past range of *Wollemia* and/or *Agathis* conifers that produce this pollen type and adds to the growing paleobotanical evidence for extensive trans-Antarctic interchange between Patagonia and Australia during the globally warm early Paleogene.

## Geological Setting and Age Control

### Ethics Statement

All necessary permits were obtained for the described study, which complied with all relevant regulations. Permits were issued by the Secretaría de Estado de Cultura de la Provincia de Santa Cruz, Argentina.

**Figure 1:**
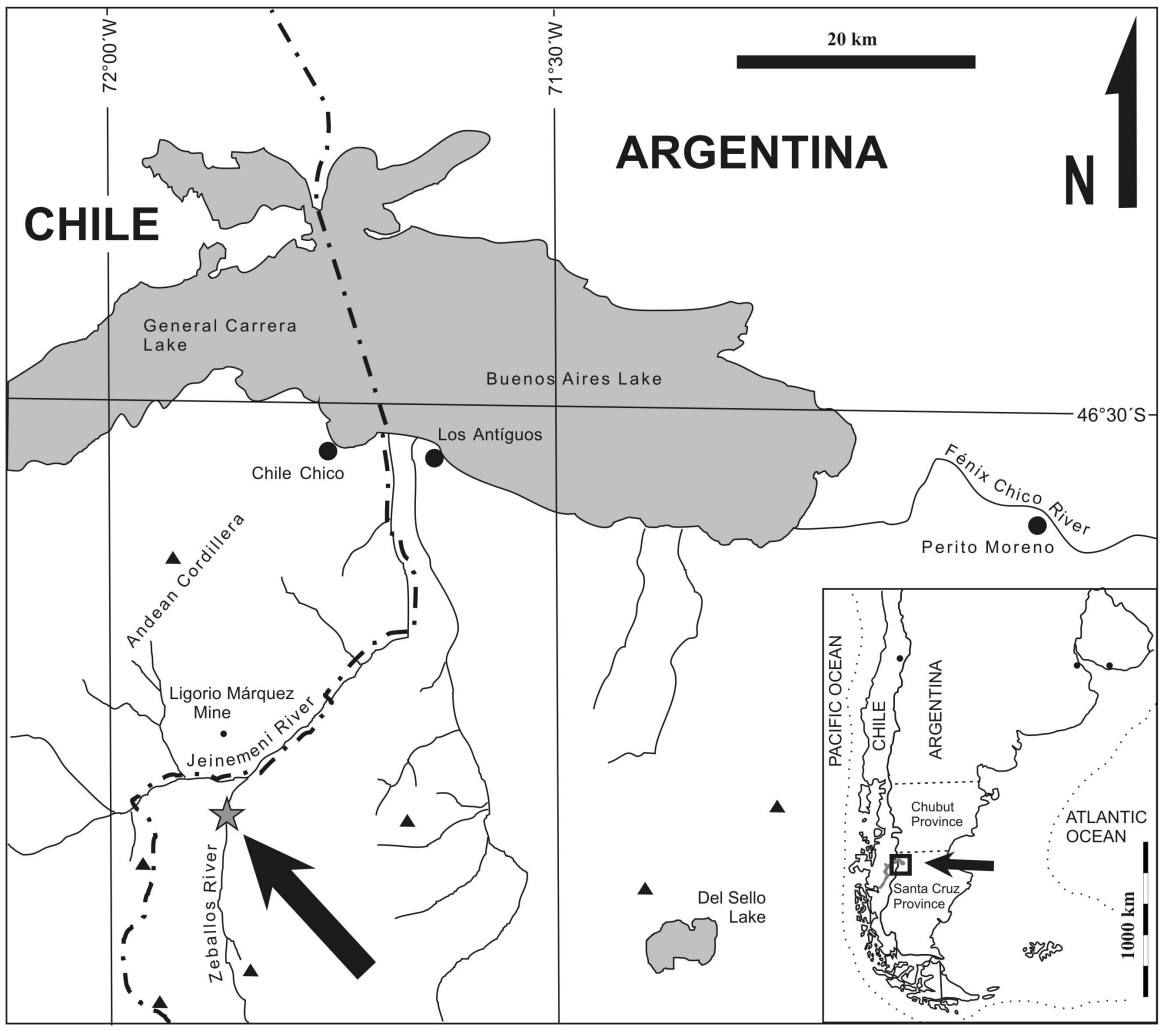
Map of study area. The new fossil plant locality occurs along the Río Zeballos (arrow, star) in Santa Cruz Province, Argentina. Also shown is the previously reported type locality of the Ligorio Márquez Formation [26], the Ligorio Márquez coal mine in XI Región, Chile.

The *Dilwynites* specimens reported here came from an isolated, newly recognized, streamcut outcrop of the Ligorio Márquez Formation in Santa Cruz Province, Patagonia, Argentina, located along the Río Zeballos and 36 km south-southwest of the town of Los Antiguos (Fig. 1). Precise locality data are available on request from AI, PW, or Museo Padre Jesús Molina, Río Gallegos, Santa Cruz, Argentina (MPM), where material is stored. The Ligorio Márquez Formation, previously studied only on the Chilean side of the nearby border [25]–[27], comprises a sequence of coastal floodplain, fluvial and mire facies deposited in a foreland basin (the Ligorio Márquez Basin) that subsequently was uplifted by compressional Andean tectonic activity during and since the early Miocene [28], [29]. The material studied here was derived from a ∼0.5 m thick carbonaceous shale bed containing abundant fossil leaves (under separate study), from a probable coastal swamp.

The local exposure of the Ligorio Márquez Formation lies unconformably above the Cretaceous Río Tarde Formation and is itself overlain unconformably by marine rocks of the Centinela Formation [30]–[32]. Stratigraphic correlation of the fossil locality is extremely difficult due to limited outcrop area and local cover. All radioisotopic ages listed below are as originally reported and would need recalibration with new constants and reanalyses with updated methods for any detailed analysis.

^40^K/^39^Ar dates derived from ash beds at the top of the Río Tarde Formation at Lago Posadas (100 km south of our study area) were 97.1±3.8 and 99.1±5.6 Ma [33]. Whole-rock ^40^Ar/^39^Ar analyses of an altered ash bed from the Centinela Formation south of Calafate (420 km to the south) yielded a range of ages with large scatter, from which the authors suggested a best estimate of 46±2 Ma [34]. This suggests a middle Eocene minimum age for the Centinela transgression in western Santa Cruz and thus of the fossil flora studied here. However, it is not certain that the Centinela exposures in our study area are correlative with those dated [34]. Our attempts to date basaltic intrusions superposed above the Río Zeballos locality did not yield informative analytical results (B. Jicha, pers. comm. 2012), although some basic intrusions in the area are associated with the Los Antiguos Teschenite and the Posadas Formation. The Los Antiguos Teschenite intrudes the Río Tarde Formation and yielded early–middle Eocene ^40^K/^39^Ar ages of 46±3 and 48±4 Ma [35]–[38]. The basalts from the Posadas Formation were dated on the Argentinean side to 43.5±7 Ma [33]. At the Chilean type locality [26], the Ligorio Márquez coal mine (Fig. 1), the Ligorio Márquez Formation comprises a c. 55 m thick succession of alternating subhorizontal beds of mudstones, quartz-rich sandstones and thin coals, unconformably underlain by Lower Cretaceous tuffs, the Flamencos Tuffs, and overlain by basalts with a ^40^K/^39^Ar age on plagioclase of 47.6±0.78 Ma above the mine [27] but which elsewhere range in age from c. 57 Ma to c. 41 Ma [26], [27], [38], [39].

In summary, all the geochronologic evidence, while greatly in need of revision, is most consistent with an early middle Eocene (Lutetian) minimum age for the fossil flora at Río Zeballos. This inference is best supported by the recent ^40^K/^39^Ar dates of 47.6±0.78 Ma, analyzed from units that immediately overlie the type strata of the Ligorio Márquez Formation in Chile [27].

**Figure 2:**
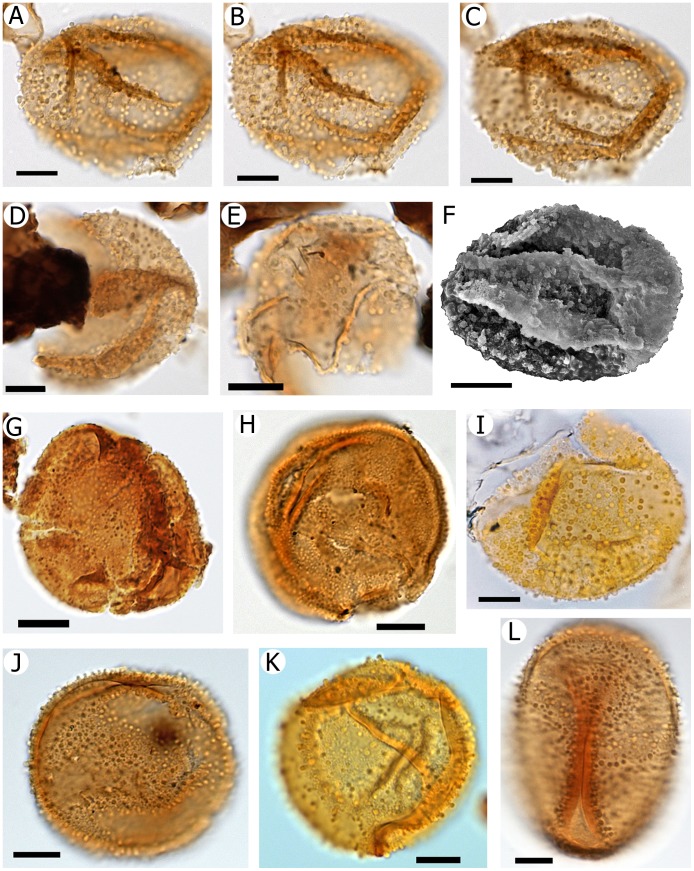
Microscope images of *Dilwynites* spp. (A–K) and *Agathis* pollen (L). .A–F, *Dilwynites* sp. cf. *D. tuberculatus* from the Río Zeballos locality, Ligorio Márquez Formation, Santa Cruz, Argentina. A–C, single grain, showing details including clavae/gemmae at three focal planes. D–F, other specimens (F is a scanning electron microscope image). G, H, *Dilwynites granulatus* from Australia showing granulate ornamentation. G, Ti-tree Basin, Northern Territory (early Eocene). H, Frome Embayment, South Australia (Miocene). I–K, *Dilwynites tuberculatus* from Australia, showing sculptural elements that are similar to those of the Río Zeballos specimens (A–F), and which are more pronounced and more widely spaced than in *D. granulatus* (G, H). I, Cethana, Tasmania (early Oligocene). J, Ti-tree Basin, Northern Territory (early Eocene). K, Lowana Rd, Tasmania (early Eocene). L, *Agathis ovata* recent specimen from Mts. des Koghis (Queensland Herbarium specimen AQ 391532: W.G. Ziarnik 34), New Caledonia. Note strong similarity to *Dilwynites* spp. Scale bars: 10 µm.

**Figure 3:**
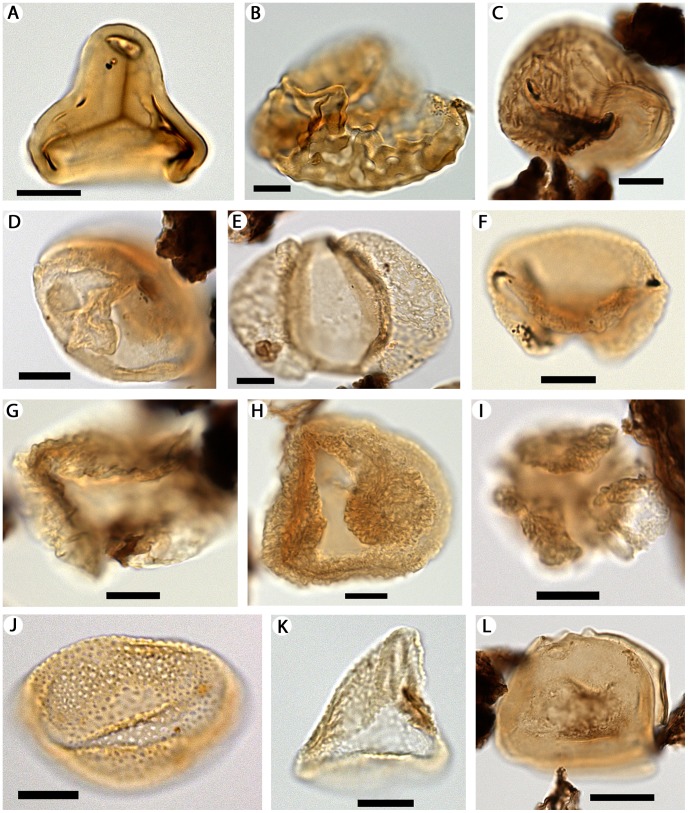
Microscope images of cryptogam spores (A–C), other gymnosperm pollen (D–I) and monocot pollen (J–L) from the Río Zeballos locality. Suggested extant affinities, if known, are shown in parentheses. A, *Cyathidites* sp. (Cyatheaceae). B, *Ischyosporites areapunctata* (Stuchlik) Barreda (Dicksoniaceae). C, *Reboulisporites fuegiensis* Zamaloa & E.J. Romero (Aytoniaceae). D, *Phyllocladidites mawsonii* Cookson ex Couper (*Lagarostrobos*). E, *Podocarpidites marwickii* Couper (*Podocarpus*/*Prumnopitys*). F, *Podosporites microsaccatus* (Couper) M.E. Dettmann (*Microcachrys*). G, *Dacrycarpites australiensis* Cookson & K.M. Pike (*Dacrycarpus*). H, *Dacrydiumites florinii* Cookson & K.M. Pike var. (*Dacrydium*). I, *Microcachryidites antarcticus* Cookson (*Microcachrys*). J, *Liliacidites* cf. *L.* r*egularis* Archangelsky (Liliaceae). K, *Luminidites* sp. (Agavaceae). L, *Proxapertites* sp. (Araceae/Arecaceae). Scale bars: A–K, 10 µm; L, 20 µm.

**Figure 4:**
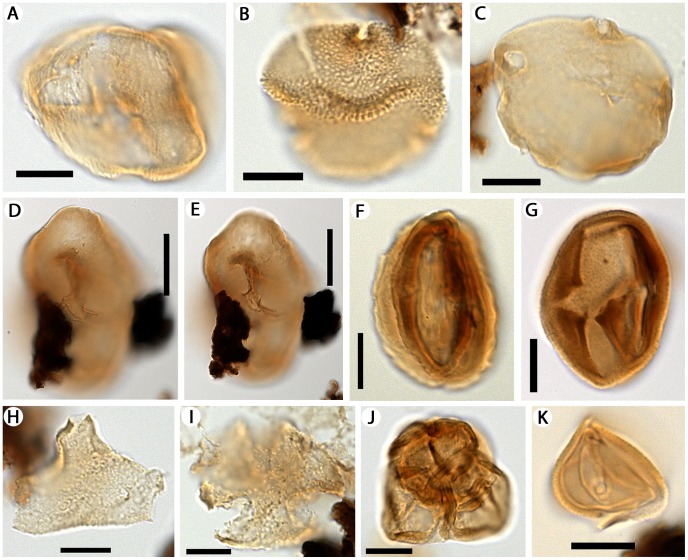
Microscope images of dicot pollen from the Río Zeballos locality. Suggested extant affinities, if known, are shown in parentheses. A, *Ailanthipites* sp. (Anacardiaceae). B, *Bombacacidites* sp. (bombacoid Malvaceae). C, *Stephanocolpites* sp. (cf. Haloragaceae). D, E, Triprojectacites group cf. *Integricorpus* sp. (at two focal planes). F, *Mutisiapollis* sp. (Asteraceae). G, *Tricolporites* sp. H, *Proteacidites* sp. (Proteaceae). I, *Spinitricolpites* sp. J, *Ericipites microverrucatus* (Ericales). K, *Schizocolpus* sp. (Didymelaceae). Scale bars: A–C, F–K, 10 µm; D, E, 20 µm.

The palynoflora so far studied at the Río Zeballos site comprises a total of 25–30 taxa of cryptogam spores and gymnosperm and angiosperm pollen (Figs. 2A–F, 3, 4). Podocarpaceous gymnosperms include *Dacrycarpites australiensis* (*Dacrycarpus*; Fig. 3G), *Dacrydiumidites florinii* (*Dacrydium*; Fig. 3H), *Phyllocladidites mawsonii* (*Lagarostrobos*; Fig. 3D), *Microcachryidites antarcticus* (*Microcachrys*; Fig. 3I), and *Podosporites microsaccatus* (*Microcachrys*; Fig. 3E). In general, these taxa are indicative of regional microtherm to mesotherm rainforest vegetation [40]. Angiosperm pollen (Figs. 3J–L, 4) includes mesotherm to possible megatherm taxa such as *Proxapertites* sp. (Araceae/Arecaceae; Fig. 3L) and *Bombacacidites* (bombacoid Malvaceae; Fig. 4B). Pollen of the microtherm to mesotherm rainforest genus *Nothofagus* was not recorded. This absence of a taxon that is usually very abundant when present, from an otherwise diverse palynoflora, also implies that our samples are most likely to be early Eocene in age, an interval that in Patagonia has long been noted to lack *Nothofagus*, and certainly no younger than middle Eocene (e.g., [41]–[46]).

The macroflora is currently the subject of a separate study, but so far, *Dacrycarpus* (presumably corresponding to the *Dacrycarpites australiensis* pollen) has been identified as well as a possible cycad and many angiosperms, including several species of Lauraceae, a family that is also well represented in Chilean samples of the Ligorio Márquez Formation [47]. In accord with the palynological data, *Nothofagus* macrofossils are absent. *Dacrycarpus* macrofossils are otherwise known in Patagonia only from early and middle Eocene strata [48], although *Dacrycarpites australiensis* pollen is present in the region until the Miocene [49].

Although more study is clearly needed, the combined geological and paleobotanical evidence suggests an early Eocene age for the Río Zeballos material studied here, and most conservatively, its age lies within the late Paleocene to early middle Eocene interval.

## Results

### Systematic Paleontology

Turma: Aletes.

Subturma: Azonaletes.

Infraturma: Subpilonapiti.

Genus: *Dilwynites* W.K. Harris, 1965 [10].

*Dilwynites* sp. cf. *D. tuberculatus* W.K. Harris 1965 [10].

#### Description

Monad, apolar; inaperturate, spheroidal but usually flattened and/or folded; exine thin, less than 1 µm thick, densely ornamented with irregularly-spaced clavae c. 1–2 µm in diameter and height, areas between the sculptural elements apparently psilate; 36–(49)–52 µm in maximum diameter (10 specimens measured).

#### Illustrations

Figs. 2A–F.

#### Material and referred specimens

Ligorio Márquez Formation carbonaceous shale from the Río Zeballos locality, Santa Cruz, Argentina. Mounted specimens can be found on slide MPM-PB-14715.

#### Age

Late Paleocene to early middle Eocene, and most probably early Eocene.

#### Distribution

So far known only from the Río Zeballos locality, Santa Cruz, Patagonia, Argentina.

#### Affinity

*Wollemia*/*Agathis* (Araucariaceae).

#### Remarks

The specimens from the Ligorio Márquez Formation differ from *Dilwynites granulatus* (Figs. 2G, H) and *D. tuberculatus* (Figs. 2I–K), described from the Danian to Selandian (early to middle Paleocene) Pebble Point Formation, South Australia [10], in that the coarse ornamentation of the fossils studied here consists of clavae (which may appear as gemmae in poorly preserved specimens) rather than granula and verrucae-tuberculae, respectively. The exines of the new fossils are also thinner. Pseudo-laesurae created by folding superficially resemble the trilete apertures on baculate-rugulate spores assigned to *Baculatisporites*, e.g. *B. turbioensis* Archangelsky in Argentina (see Fig. 5A in [50]). Lauraceae pollen is similar to that of Araucariaceae in being spheroidal and inaperturate, but it typically differs from *Dilwynites* in having ornamentation that consists of regularly-spaced, sharply pointed echinae, spinulae or foveolae (see e.g., Plate 33, images 391–393 in [51]). Moreover, it is well known that Lauraceae pollen is generally absent from fossil assemblages, mostly because it has only very thin exine with little sporopollenin [52], and probably very low production [53].

Here, we infer that the *Dilwynites* specimens from the Ligorio Márquez Formation potentially indicate the past presence of *Wollemia* in Patagonia because the specimens closely resemble pollen of *W*. *nobilis* [6], [11], [12]. Alternatively, the fossil pollen could be attributed to *Agathis* because it has recently become apparent that several extant species of *Agathis* produce grains ornamented with relatively coarse granules (M.K. Macphail, unpublished data) and thus would be accommodated within *Dilwynites* if found as fossils. Examples of *Agathis* species producing this type of pollen include the New Caledonian *A. ovata* (Vieill.) Warb. (Fig. 2L) and *A. moorei* (Lindl.) Mast. Other extant Araucariaceae (and in particular *Araucaria*) pollen most obviously differ in having much less prominent surface ornamentation [12]. Further refinement of relationships between *Dilwynites* and extant taxa may be possible following more detailed comparisons.

## Discussion

The Ligorio Márquez Formation specimens of *Dilwynites* are the first known record of *Wollemia*-type pollen in South America. It is uncertain whether the newly recognized Patagonian clavate morphotype of *Dilwynites* represents a new species, given the wide range of variation observed in the granulate sculptural elements characterizing *D*. *granulatus* and the baculate to tuberculate sculptural elements characterizing *D*. *tuberculatus* populations in Australia (see Figs. 2G–K). The same is true from preliminary observations of other gymnosperm pollen taxa in the Ligorio Márquez Formation sample, which differ from the ranges of morphologies observed in Australian populations and those recorded from the Falkland (Malvinas) Islands (compare Figs. 3D–I with, e.g., Fig. 21 in [54]). A not unreasonable conclusion is that degrees of geographic differentiation occurred over the very long distances of these plants’ ancient ranges.

Both *Araucaria* and *Agathis* have substantial macrofossil records in the Southern Hemisphere, but there is no strong macrofossil evidence for *Wollemia* (reviews [55]–[59]). *Araucaria* occurs extensively in Patagonia from the Early Jurassic to present, in West Antarctica from the Jurassic or Early Cretaceous to Eocene, and in Australia and New Zealand from the Early Cretaceous. The much more fragmentary *Agathis* record formerly came only from Cenozoic Australia and New Zealand, but abundant macrofossil *Agathis* specimens from the early and middle Eocene of northwestern Patagonia are now being described [60]; these include pollen cones, but pollen grains are not preserved within them. Reliable macrofossil evidence for *Agathis* is so far unknown from the Mesozoic [7], [55], [57].

At present, the macrofossils most likely to have close affinity to *Wollemia* are leaves of *Araucarioides* from Australia and New Zealand [6], and it is especially interesting that at the early Eocene Lowana Road site in Tasmania, these leaves co-occur with relatively abundant *Dilwynites tuberculatus* pollen [61]. As stated previously, recent molecular and reproductive data resolve *Wollemia* and *Agathis* as likely sister taxa [18]–[24]. This evidence, combined with the fact that *Dilwynites* first appears in the fossil record much later (Turonian: Late Cretaceous) than *Araucaria* suggests that at least some Mesozoic fossils that cannot be assigned to *Araucaria* can now be regarded as belonging to the stem lineage of the *Agathis*+*Wollemia* clade [24]. These fossils include winged seeds and cone scales with seed detachment scars from the Early to mid-Cretaceous in southeastern Australia [6], [62], New Zealand [63] and Alexander Island, West Antarctica [64]. It should also be noted that at least some of the pollen included in the generalized, widespread form *Araucariacites australis* Cookson, which extends to the Triassic in the Southern Hemisphere, and which broadly accommodates pollen of modern *Araucaria* and many *Agathis* (e.g. [15]), could have been produced by extinct close relatives of *Agathis* and *Wollemia*.

The only extant Araucariaceae in South America are *Araucaria angustifolia* (Bertol.) Kuntze, native to southern Brazil and northeastern Argentina, where it is a dominant in temperate to subtropical rainforest, and *A. araucana* (Molina) K. Koch, native to Andean central and southern Chile and western Argentina between latitudes c. 37 to 40°S, where it associates with *Nothofagus* spp. to form mixed forests above c. 600–900 m elevation. The two species are apparently the survivors of the considerably more diverse Mesozoic araucarian flora of South America, represented by wood, foliage, cone, and pollen material (e.g., [58], [65]–[68]). This flora reached its maximum diversity and dominance in Patagonia, where araucarians were often co-dominant with cheirolepidiaceous conifers [69], [70] during the Jurassic to Early Cretaceous. By the early and early middle Eocene, *Araucaria* and *Agathis* were abundant, but not diverse in Patagonia, occurring in association with crown group Podocarpaceae and Cupressaceae conifers with Australasian affinities and very diverse angiosperms [48], [60], [71], [72]. However, so far as is known, none of the previously reported South American fossil species is comparable to *Wollemia*.

Our evidence extends the geographic range of the araucarian lineage(s) that produced *Wollemia*/*Agathis* (coarsely granulate)-type pollen to South America. This is a significant contribution to the emerging biogeographic pattern for Paleogene Gondwana, where there are numerous shared extant genera known as macrofossils (e.g., *Dacrycarpus*, *Papuacedrus*, *Gymnostoma*, *Eucalyptus*) and/or microfossils from Eocene floras of both southern South America and Australia and occasionally Antarctica (e.g., [48], [60], [71]–[79]). These trans-Antarctic distributions are increasingly comparable to the numerous examples of Holarctic floral and faunal interchange during this globally warm time interval.

## Materials and Methods

Blocks of wet sediment containing abundant mummified leaves (under separate study) were collected 3–4 May, 2011 at the Argentine Ligorio Márquez Formation outcrop (Fig. 1), wrapped in plastic to minimize water loss, and temporarily stored in a large refrigerator. Sediment samples selected for palynological processing were then oven-dried. Microfossils were extracted and replicate slides prepared by M. Rueda, Paleoflora Ltd, Bucaramanga, Colombia, using standard protocols. Microfossils were examined and photographed at ANU, Canberra, Australia using a Leica Axiophot transmitted light microscope fitted with AxioVision image capturing software. Residues were also examined and microfossils photographed at La Plata University, La Plata, Argentina using a JEOL JSM-6360LV scanning electron microscope operated at 10 kV. Adobe Photoshop Elements 6.0 software was used to optimise brightness and contrast of images, and to compose figures.

## References

[1] Jones WD, Hill KD, Allen JM (1995) *Wollemia nobilis*, a new living Australian genus and species in the Araucariaceae. Telopea 6: 173–176.

[2] White ME (1981) Revision of the Talbragar Fish Bed flora (Jurassic) of New South Wales. Rec Aust Mus 33: 695–721.

[3] Bigwood AJ, Hill RS (1985) Tertiary araucarian macrofossils from Tasmania. Aust J Bot 33: 645–656.

[4] Hill RS, Bigwood AJ (1987) Tertiary gymnosperms from Tasmania: Araucariaceae. Alcheringa 11: 325–335.

[5] Pole M (1995) Late Cretaceous macrofloras of Eastern Otago, New Zealand: Gymnosperms. Aust Syst Bot 8: 1067–1106.

[6] Chambers TC, Drinnan AN, McLoughlin S (1998) Some morphological features of Wollemi Pine (*Wollemia nobilis*: Araucariaceae) and their comparison to Cretaceous plant fossils. Int J Plant Sci 159: 160–171.

[7] Hill RS, Lewis T, Carpenter RJ, Whang SS (2008) *Agathis* (Araucariaceae) macrofossils from Cainozoic sediments in south-eastern Australia. Aust Syst Bot 21: 162–177.

[8] Turner S, Bean LB, Dettmann M, McKellar J, McLoughlin S, et al (2009) Australian Jurassic sedimentary and fossil successions: current work and future prospects for marine and non-marine correlation. GFF 131: 49–70.

[9] Woodford J (2005) The Wollemi Pine: the incredible discovery of a living fossil from the age of the dinosaurs (Revised Edition). Melbourne: Text Publishing. 224 p.

[10] Harris WK (1965) Basal Tertiary microfloras from the Princetown area, Victoria, Australia. Palaeontographica B 115: 75–106.

[11] Macphail MK, Hill K, Partridge AD, Truswell EM (1995) ‘Wollemi Pine’ - old pollen records for a newly discovered genus of gymnosperms. Geol Today 11: 48–50.

[12] Dettmann ME, Jarzen DM (2000) Pollen of extant *Wollemia* (Wollemi Pine) and comparisons with pollen of other extant and fossil Araucariaceae. In: Harley MM, Morton CM, Blackmore S, editors. Pollen and spores: morphology and biology. Kew: Royal Botanic Gardens. 187–203.

[13] Gradstein FM, Ogg JG, Schmitz MD, Ogg GM, Agterberg FP, et al.. (2012) The geologic time scale 2012. Boston, USA: Elsevier. 1145 pp.

[14] Macphail M (2007) Australian palaeoclimates: Cretaceous to Tertiary – a review of palaeobotanical and related evidence to the year 2000. CRC LEME Spec Vol Open File Rep 151. 266 p.

[15] Raine JI, Mildenhall DC, Kennedy EM (2011) New Zealand pollen and spores: an illustrated catalogue. GNS Sci Misc Ser 4. http://data.gns.cri.nz/sporepollen/index.htm. Accessed 25 August 2012.

[16] Truswell EM, Macphail MK (2008) Polar forests on the edge of extinction: what does the fossil pollen and spore evidence say? Aust Syst Bot 22: 57–106.

[17] Askin RA (1990) Campanian to Paleocene spore and pollen assemblages of Seymour Island, Antarctica. Rev Palaeobot Palynol 65: 105–113.

[18] Gilmore S, Hill KD (1997) Relationships of the Wollemi pine (*Wollemia nobilis*) and a molecular phylogeny of the Araucariaceae. Telopea 7: 275–291.

[19] Stefanović S, Jager M, Deutsch J, Broutin J, Masselot M (1998) Phylogenetic relationships of conifers inferred from partial 28S rRNA gene sequences. Am J Bot 85: 688–697. 21684951

[20] Rai HS, Reeves PA, Peakall R, Olmstead RG, Graham SW (2008) Inference of higher-order conifer relationships from a multi-locus plastid data set. Botany 86: 658–669.

[21] Liu N, Zhu Y, Wei ZX, Chen J, Wang QB, et al (2009) Phylogenetic relationships and divergence times of the family Araucariaceae based on the DNA sequences of eight genes. Chin Sci Bull 54: 2648–2655.

[22] Biffin E, Hill RS, Lowe AJ (2010) Did Kauri (*Agathis*: Araucariaceae) really survive the Oligocene drowning of New Zealand? Syst Biol 59: 594–602. 20530131 10.1093/sysbio/syq030

[23] Leslie AB, Beaulieu JM, Rai HS, Crane PR, Donoghue MJ, et al (2012) Hemisphere-scale differences in conifer evolutionary dynamics. Proc Natl Acad Sci USA 109: 16217–16221. 22988083 10.1073/pnas.1213621109 PMC3479534

[24] Dettmann ME, Clifford HT, Peters M (2012) *Emwadea microcarpa* gen. et sp. nov.–anatomically preserved araucarian seed cones from the Winton Formation (late Albian), western Queensland, Australia. Alcheringa 36: 217–237.

[25] Suárez M, de la Cruz R (1996) Estratigrafía y tectónica de la zona sureste del Lago General Carrera (46°30′–47° Lat.S.), Cordillera Patagónica, Chile. Actas XIII Congr Geol Argent y III Congr Explor Hidrocarb I: 425–432.

[26] Suárez M, de la Cruz R, Troncoso A (2000) Tropical/subtropical Upper Paleocene-Lower Eocene fluvial deposits in eastern central Patagonia, Chile (46°45′S). J S Am Earth Sci 13: 527–536.

[27] Yabe A, Uemura K, Nishida H (2006) Geological notes on plant localities of the Ligorio Márquez Formation, central Patagonia, Chile. In: Nishida H, editor. Post-Cretaceous floristic changes in southern Patagonia, Chile. Tokyo: Chuo University. 29–35.

[28] Suárez M, De La Cruz R (2000) Tectonics in the eastern central Patagonian Cordillera (45°30′–47°30′S). J Geol Soc Lond 157: 995–1001.

[29] Blisniuk PM, Stern LB, Chamberlian CP, Idleman B, Zeitler PK (2005) Climatic and ecologic change during Miocene surface uplift in the Southern Patagonian Andes. Earth Planet Sci Lett 230: 125–142.

[30] Ugarte FRE (1956) El Grupo de Río Zeballos en el flanco occidental de la Meseta de Buenos Aires (Provincia de Santa Cruz). Rev Asoc Geol Argent 11: 202–216.

[31] Escosteguy L, Franchi M, Dal Molín C (2001) Formación Ligorio Márquez (Paleoceno superior–Eoceno inferior) en el Río Zeballos, Provincia de Santa Cruz, Argentina. 11°Congr Latinoam Geol y 3°Congr Urug Geol, Montevideo, Abstr.: 10.

[32] Escosteguy L, Dal Molín C, Franchi M, Geuna S, Lapido O (2003) Hoja geológica 4772-II, Lago Buenos Aires. Serv Geol Min Argent Bol 339.

[33] Ramos V, Drake R (1987) Edad y significado tectónico de la Formación Río Tarde (Cretácico), Lago Posadas, provincia de Santa Cruz. Actas 10°Congr Geol Argent 1: 143–147.

[34] Casadío S, Feldmann RM, Foland KA (2000) ^40^Ar/^39^Ar age and oxygen isotope temperature of the Centinela Formation, southwestern Argentina: an Eocene age for crustacean-rich “Patagonian” beds. J S Am Earth Sci 13: 123–132.

[35] Busteros AG, Lapido OR (1983) Rocas básicas en la vertiente noroccidental de la meseta del Lago Buenos Aires, provincia de Santa Cruz. Rev Asoc Geol Argent 38: 427–436.

[36] Linares E, González R (1990) Catálogo de edades radimétricas de la República Argentina, años 1957–1987. Publ Espec Asoc Geol Argent B 19: 1–628.

[37] Ramos VA, Mahlburg Kay S, Sacomani L (1994) La dacita Puesto Nuevo y otras rocas magmáticas (Cordillera Patagónica Austral): colisión de una dorsal oceánica Cretácica. VII°Congr Geol Chil, Concepción, Actas 2: 1172–1176.

[38] Charrier R, Linares E, Niemeyer H, Skarmeta J (1979) K/Ar ages of basalt flows of the Meseta Buenos Aires in southern Chile and their relation to the southeast Pacific triple junction. Geology 7: 436–439.

[39] Petford N, Cheadle M, Barreiro B (1996) Age and origin of southern Patagonian flood basalts, Chile Chico region (46°45′S). ISAG 96: Symp Int Géodyn Andin, St. Malo, France, 629–632.

[40] Macphail MK, Alley N, Truswell EM, Sluiter IR (1994). Early Tertiary vegetation: evidence from pollen and spores. In: Hill RS, editor. Australian vegetation history: Cretaceous to Recent. Cambridge: Cambridge University Press. 189–261.

[41] Okuda M, Nishida H, Uemura K, Yabe A (2006) Paleocene/Eocene pollen assemblages from the Ligorio Márquez Formation, Central Patagonia, XI Región, Chile. In: Nishida H, editor. Post-Cretaceous floristic changes in southern Patagonia, Chile. Tokyo: Chuo University. 37–43.

[42] Quattrocchio M (2006) Palynology and palaeocommunities of the Paleogene of Argentina. Rev Bras Paleontol 9: 101–108.

[43] Barreda V, Palazzesi L (2007) Patagonian vegetation turnovers during the Paleogene–Early Neogene: origins of arid-adapted floras. Bot Rev 73: 31–50.

[44] Melendi DL, Scafati LH, Volkheimer W (2003) Palynostratigraphy of the Paleogene Huitrera Formation in N-W Patagonia, Argentina. Neues Jahrb Geol Palaontol Abh 228: 205–273.

[45] Troncoso A, Romero EJ (1998) Evolución de las comunidades florísticas en el extremo sur de Sudamérica durante el Cenofitico. Monogr Syst Bot Mo Bot Gard 68: 149–172.

[46] Wilf P, Singer BS, Zamaloa MC, Johnson KR, Cúneo NR (2010) Early Eocene ^40^Ar/^39^Ar age for the Pampa de Jones plant, frog, and insect biota (Huitrera Formation, Neuquén Province, Patagonia, Argentina). Ameghiniana 47: 207–216.

[47] Troncoso A, Suárez M, de la Cruz R, Palma-Heldt S (2002) Paleoflora de la Formación Ligorio Márquez (XI Región, Chile) en su localidad tipo: sistemática, edad e implicancias paleoclimáticas. Rev Geol Chil 29: 113–135.

[48] Wilf P (2012) Rainforest conifers of Eocene Patagonia: attached cones and foliage of the extant Southeast Asian and Australasian genus *Dacrycarpus* (Podocarpaceae). Am J Bot 99: 562–584. 22334450 10.3732/ajb.1100367

[49] Barreda VD (1996) Bioestratigrafía de polen y esporas de la Formación Chenque, Oligoceno tardío?–Mioceno de las Provincias de Chubut y Santa Cruz, Patagonia, Argentina. Ameghiniana 33: 35–56.

[50] Quattrocchio M, Martínez M, Asensio MA, Cormou ME, Olivero DE (2012) Palynology of the El Foyel Group (Paleogene), Ñirihuau Basin, Argentina. Rev Bras Paleontol 15: 67–84.

[51] Heusser CJ (1971) Pollen and spores of Chile. Tucson: University of Arizona Press. 167 p.

[52] Erdtman G (1952) Pollen morphology and plant taxonomy: angiosperms. Stockholm: Almqvist & Wiksell. 539 p.

[53] Macphail MK (1980) Fossil and modern *Beilschmiedia* (Lauraceae) pollen in New Zealand. NZ J Bot 18: 453–457.

[54] Macphail MK, Cantrill D (2006) Age and implications of the Forest Bed, Falkland Islands, southwest Atlantic Ocean: evidence from fossil pollen and spores. Palaeogeogr Palaeoclimatol Palaeoecol 240: 602–629.

[55] Hill RS, Brodribb TJ (1999) Turner Review No. 2: Southern conifers in time and space. Aust J Bot 47: 639–696.

[56] Dettmann ME, Clifford HT (2005) Biogeography of Araucariaceae. In: Occasional Publication Dargavel J, editor. Australia and New Zealand forest histories: araucarian forests. Australian Forest History Society Inc. 2: 1–9.

[57] Pole MS (2008) The record of Araucariaceae macrofossils in New Zealand. Alcheringa 32: 405–426.

[58] Panti C, Pujana RR, Zamaloa MC, Romero EJ (2012) Araucariaceae macrofossil record from South America and Antarctica. Alcheringa 36: 1–22.

[59] Kunzmann L (2007) Araucariaceae (Pinopsida): aspects in palaeobiogeography and palaeodiversity in the Mesozoic. Zoologischer Anzeiger 246: 257–277.

[60] Wilf P, Escapa IH, Cúneo NR (2012) Eocene rainforest conifers of the Patagonian fire lakes. Bot Soc Am 2012, Columbus, Ohio, Abstr: 175.

[61] Carpenter RJ, Jordan GJ, Macphail MK, Hill RS (2012) Near-tropical Early Eocene terrestrial temperatures at the Australo-Antarctic margin, western Tasmania. Geology 40: 267–270.

[62] Drinnan AN, Chambers TC (1986) Flora of the Lower Cretaceous Koonwarra fossil bed (Korumburra group), south Gippsland, Victoria. Assoc Australas Palaeontol Mem 3: 1–77.

[63] Cantrill DJ, Raine JI (2006) *Wairarapaia mildenhallii* gen. et sp. nov., a new araucarian cone related to *Wollemia* from the Cretaceous (Albian–Cenomanian) of New Zealand. Int J Plant Sci 167: 1259–1269.

[64] Cantrill DJ, Falcon-Lang HJ (2001) Cretaceous (Late Albian) coniferales of Alexander Island, Antarctica. 2. Leaves, reproductive structures and roots. Rev Palaeobot Palynol 115: 119–145. 11440766 10.1016/s0034-6667(01)00053-7

[65] Del Fueyo GM, Archangelsky S, Archangelsky A (2012) An ultrastructural study of the araucarian pollen grain *Cyclusphaera radiata* Archangelsky from the Albian of Patagonia. Rev Palaeobot Palynol 173: 57–67.

[66] Spegazzini C (1924) Coniferales fósiles Patagónicas. An Soc Cient Argent 97: 125–139.

[67] Stockey RA (1975) Seeds and embryos of *Araucaria mirabilis* . Am J Bot 62: 856–868.

[68] Del Fueyo GM, Archangelsky S (2005) A new araucarian pollen cone with in situ *Cyclusphaera* Elsik from the Aptian of Patagonia, Argentina. Cretac Res 26: 757–768.

[69] Escapa IH, Rothwell GW, Stockey RA, Cúneo NR (2012) Seed cone anatomy of Cheirolepidiaceae (Coniferales): reinterpreting *Pararaucaria patagonica* Wieland. Am J Bot 99: 1058–1068. 22665438 10.3732/ajb.1100544

[70] Escapa IH, Cúneo NR, Rothwell GW, Stockey RA (2013) *Pararaucaria delfueyoi* sp. nov. from the Late Jurassic Cañadón Calcáreo Formation, Chubut, Argentina: insights into the evolution of the Cheirolepidiaceae. Int J Plant Sci 174: 458–470.

[71] Wilf P, Little SA, Iglesias A, Zamaloa MC, Gandolfo MA, et al (2009) *Papuacedrus* (Cupressaceae) in Eocene Patagonia: a new fossil link to Australasian rainforests. Am J Bot 96: 2031–2047. 21622323 10.3732/ajb.0900085

[72] Wilf P, Johnson KR, Cúneo NR, Smith ME, Singer BS, et al (2005) Eocene plant diversity at Laguna del Hunco and Río Pichileufú, Patagonia, Argentina. Am Nat 165: 634–650. 15937744 10.1086/430055

[73] Hill RS, Carpenter RJ (1991) Extensive past distributions for major Gondwanic floral elements: macrofossil evidence. Pap Proc Roy Soc Tas 125: 239–247.

[74] Zamaloa MC, Gandolfo MA, González CC, Cunéo NR, Wilf P (2006) Casuarinaceae from the Eocene of Patagonia, Argentina. Int J Plant Sci 167: 1279–1289.

[75] Gandolfo MA, Hermsen EJ, Zamaloa MC, Nixon KC, González CC, et al (2011) Oldest known *Eucalyptus* macrofossils are from South America. PLoS One 6: e21084. 21738605 10.1371/journal.pone.0021084 PMC3125177

[76] Carpenter RJ (2012) Proteaceae leaf fossils: phylogeny, diversity, ecology and austral distributions. Bot Rev 78: 261–287.

[77] Hermsen EJ, Gandolfo MA, Zamaloa MC (2012) The fossil record of *Eucalyptus* in Patagonia. Am J Bot 99: 1356–1374. 22859652 10.3732/ajb.1200025

[78] Macphail MK, Partridge AD (2012) First fossil pollen record of *Auriculiidites* Elsik, 1964 in Australia. Alcheringa 36: 283–286.

[79] Wilf P, Cúneo NR, Escapa IH, Pol D, Woodburne MO (2013) Splendid and seldom isolated: the paleobiogeography of Patagonia. Ann Rev Earth Planet Sci 41 (in press): doi: 10.1146/annurev-earth-050212-124217.

